# Quantifying the use of bioresources for promoting their sharing in scientific research

**DOI:** 10.1186/2047-217X-2-7

**Published:** 2013-05-01

**Authors:** Laurence Mabile, Raymond Dalgleish, Gudmundur A Thorisson, Mylène Deschênes, Robert Hewitt, Jane Carpenter, Elena Bravo, Mirella Filocamo, Pierre Antoine Gourraud, Jennifer R Harris, Paul Hofman, Francine Kauffmann, Maria Angeles Muñoz-Fernàndez, Markus Pasterk, Anne Cambon-Thomsen

**Affiliations:** 1 Epidémiologie et analyses en santé publique, Faculté de médecine, UMR1027 INSERM-Université de Toulouse III, 37 allées Jules Guesde, Toulouse Cedex 7, F-31073, France; 2 UMR1027 INSERM-Université de Toulouse III-Paul Sabatier, 118 Route de Narbonne, Toulouse Cedex 9, 31062, France; 3 Department of Genetics, University of Leicester, University Road, Leicester, LE1 7RH, UK; 4 Faculty of Life and Environmental Sciences, University of Iceland, Sturlugata 7, Reykjavik, 101, Iceland; 5 P3G, 2155 Guy Street Montreal, Quebec, H3H 2R9, Canada; 6 European, African & Middle Eastern Society for Biopreservation and Biobanking, 20 Boulevard du Roi René, Aix-en-Provence, 13100, France; 7 Breast Cancer Tissue Bank, University of Sydney, Darcy Road Westmead, Sydney, NSW 2145, Australia; 8 Istituto Superiore di Sanità, Department of Cellular Biology and Neuroscience, 299 Viale Regina Elena, Rome, 00161, Italy; 9 Istituto G. Gaslini, Lab Diagnosi Pre-Postnatale Malattie Metaboliche, L.go G. Gaslini, Genoa, 16147, Italy; 10 Department of Neurology, Mission Bay campus, University of California, 675 Nelson Rising Lane, San Francisco, CA, 94158, USA; 11 The Norwegian Institute of Public Health, Division of Epidemiology, PO Box 4404, Oslo, Nydalen, N-0403, Norway; 12 “Cancéropôle PACA Biobank”, Centre Hospitalo-Universitaire Pasteur, 30 Avenue de la Voie Romaine, Nice, 06000, France; 13 UMR1018 INSERM, Centre de Recherche en Epidémiologie et Santé des Populations, 16 avenue Paul Vaillant Couturier, Villejuif Cedex, 94807, France; 14 UMR1018 INSERM-Université Paris Sud, 16 avenue Paul Vaillant Couturier, Villejuif Cedex, 94807, France; 15 HIV HGM Spanish Biobank, C/Dr. Esquerdo, Madrid, 46.28007, Spain; 16 International Prevention Research Institute, 95 Cours Lafayette, Lyon, 69006, France

**Keywords:** Data sharing, Bioresource, Biobank, Identifier, Metrics, Traceability, Impact factor, Biology, Science policy, Open data

## Abstract

An increasing portion of biomedical research relies on the use of biobanks and databases. Sharing of such resources is essential for optimizing knowledge production. A major obstacle for sharing bioresources is the lack of recognition for the efforts involved in establishing, maintaining and sharing them, due to, in particular, the absence of adequate tools. Increasing demands on biobanks and databases to improve access should be complemented with efforts of end-users to recognize and acknowledge these resources. An appropriate set of tools must be developed and implemented to measure this impact.

To address this issue we propose to measure the use in research of such bioresources as a value of their impact, leading to create an indicator: Bioresource Research Impact Factor (BRIF). Key elements to be assessed are: defining obstacles to sharing samples and data, choosing adequate identifier for bioresources, identifying and weighing parameters to be considered in the metrics, analyzing the role of journal guidelines and policies for resource citing and referencing, assessing policies for resource access and sharing and their influence on bioresource use. This work allows us to propose a framework and foundations for the operational development of BRIF that still requires input from stakeholders within the biomedical community.

## Review

### Bioresources as key players in biomedical research

A growing portion of research relies on using sample collections and databases [1]. Sharing such resources is essential for optimizing knowledge production. This is especially true in biological and medical sciences with the development of large-scale biology in the –omics era [2]. The size and complexity of the collections needed to promote translational research typically extends far beyond the scope of individual research projects and the need to produce these valuable data is being met by contemporary bioresource facilities.

Bioresources are defined as biological samples with associated data (medical/epidemiological, social), and databases independent of physical samples, and other biomolecular and bioinformatics research tools (Table 1). A commitment to share the information content of bioresources with the research community is paramount to advancing translational research [3,4]. The 2011 joint statement of 17 major national health research funders sent a powerful signal that health research resources must be shared to maximize the potential of publicly funded resources [5-9]: “Funders agree to promote greater access to and use of data in ways that are: Equitable…, Ethical…, Efficient”. Although this statement gives a vision, principles, goals and aspirations, it does not indicate any practical tool or instrument to reach the objectives. Therefore, it is important to develop incentives that will support and promote this sharing if we are to realize the vision which funders hope to encourage [10,11]. One approach is to develop a system that recognizes the specific contribution of bioresources in the development of novel scientific knowledge. Whilst promoting measures to improve access to biobanks and databases, we must also develop policies mandating end-users to recognize and acknowledge the provenance of these resources. An appropriate set of tools is needed to implement such policies. Some tools currently exist, but an insufficient level of coordination and systematic implementation makes it difficult to see their positive impact on the overall organization of health research activities [6,12].

**Table 1:** Definitions


***Designation***	***Definition***
**Biospecimen**	A quantity of tissue, blood, urine, or other human-derived material. A biospecimen can comprise subcellular structures, cells, tissue (e.g. bone, muscle, connective tissue, and skin), organs (e.g., liver, bladder, heart, and kidney), blood, gametes (sperm and ova), embryos, fetal tissue, and waste (urine, feces, sweat, hair and nail clippings, shed epithelial cells, and placenta). Portions or aliquots of a biospecimen are referred to as samples (NCI* Best Practices working definition).
**Annotation**	Database information designed to capture experimental or inferential results. Often referring to annotation of sequence data. Experimental annotation is supported by peer-reviewed wet-lab experimental evidence. Inferential annotation of sequence data is by inference (where the source molecule or its product(s) have not been the subject of direct experimentation) *(from NCBI third party Annotation database)*.
**Database**	An organized set of data or collection of files that can be used for a specified purpose (definition from *A dictionary of Epidemiology 4*^*th*^*Ed. by J.M. Last*).
**Biorepository**	An organization, place, room, or container (a physical entity) where biospecimens are stored (NCI* Best Practices working definition).
**Biological resource centres**	Service providers and repositories of the living cells, genomes of organisms, and information relating to heredity and the functions of biological systems. BRCs contain collections of culturable organisms (e.g. genomes, plasmids, viruses, cDNAs), viable but not yet culturable organisms cells and tissues, as well as databases containing molecular, physiological and structural information relevant to these collections and related bioinformatics… (from OECD*** Best Practise Guidelines for BRCs, 2007).
**Biobank**	A collection of biological material and the associated data and information stored in an organised system, for a population or a large subset of a population (OECD** Glossary of statistical terms).
**Biospecimen resource**	A collection of human specimens and associated data for research purposes, the physical entity in which the collection is stored, and all associated processes and policies. Biospecimen resources vary considerably, ranging from formal institutions to informal collections in a researcher’s freezer (*NCI Best Practices* working definition).
**Bioresources**	Biological samples with associated data (medical/epidemiological, social), databases independent of physical samples and other biomolecular and bioinformatics research tools (*BRIF group* working definition).

^*^From National Cancer Institute Best Practices for Biospecimen Resources.

^**^ National Center for Biotechnology Information.

^***^Organisation for Economic Co-operation and Development.

### The BRIF initiative

The concept of a Bioresource Research Impact Factor (BRIF) was introduced in 2003 [13], and later further developed [14,15]. The BRIF initiative was set up to construct an adequate framework and provide a set of tools that will allow an objective measure of the actual research utilization of bioresources as a significant component for establishing their reliability and sustainability. An international working group was established to develop the BRIF, consisting of 134 members from 22 countries, most of whom are either European (86) or North-American (31). This group was further divided into five relevant thematic sub-groups: i) BRIF and digital identifiers, ii) BRIF parameters, iii) BRIF in sharing policies, iv) BRIF and journal editors and v) BRIF dissemination [16]. Key issues from the dedicated sub-groups’ work and the first two BRIF workshops are reported here.

### Constructing a quantitative tool to evaluate the impact of a bioresource on research

The BRIF will be modeled, to some degree, on the Journal Impact Factor (JIF) [17], and will provide guidance and methodology for optimizing recognition of bioresources, their use and their sharing at an international level. Such a tool could be used much more systematically than “reputation” for evaluating the activity of a bioresource over time. When taken into account in assessing ‘researchers/contributors’ scientific contribution, this should increase the use and sharing of bioresources, where in which a virtuous circle would occur: the highest is the quality, the most frequent will be the solicitations; the more one shares, the more one’s impact increases, and the more one is inclined to share. Although this concept can be valid for any kind of bioresource, we focus first on bioresources of human origin.

#### Stakeholders in bioresources sharing

**Figure 1:**
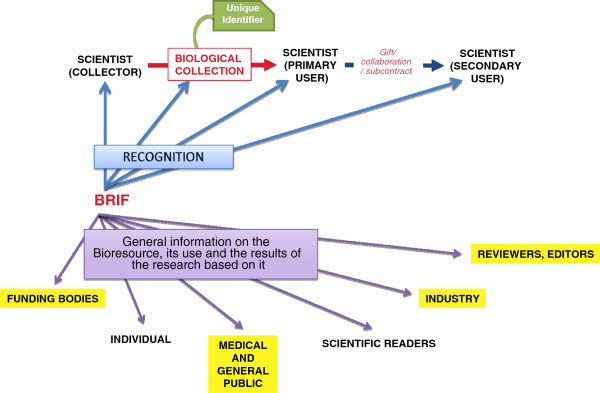
**Actors involved in the complex world of bioresources.** The upper panel (blue arrows) exhibits the chain of production and sharing of bioresources. The lower panel (purple arrows) shows the various stakeholders involved. The blue box represents the recognition needs for the upper panel and the purple box the information needs of stakeholders. BRIF that bridges the two boxes represents the tool to link these various dimensions.

BRIF aims to be a quantitative indicator filling a gap in the complex environment of scientific production assessment. Its implementation thus depends on its ability to meet the requirements of multiple stakeholders and to integrate with an already existing system of practices and parameters. BRIF could effectively enable traceability, thus being useful for all actors involved in the complex world of bioresources, from the initial collector(s) or initiator(s) to the scientific primary or secondary user(s) on the one hand; to funding bodies, the general public, scientific readers, industry and editors on the other hand. Stakeholders would benefit from the BRIF through the recognition it will generate or through the information it will offer about the bioresource, its use and the research results based on it (Figure 1).

#### Choosing adequate digital identifier schemes

The main difficulties in providing the most reliable assessment of appropriate biobank usage relates to identification and variable ways of acknowledging bioresources (Table 2). The process of tracking publications and quantifying their impact is not straightforward. In order to track the publications involving a bioresource, it is essential that researchers consistently acknowledge use of the bioresource by placing a unique and traceable identifier in all relevant publications in a defined section of the article. To some extent such tracking is already possible, provided that authors have acknowledged the bioresource or cited the bioresources’ publications, and an effort is made to find appropriate acknowledgements. In order to optimize and standardize this process, it must be automated to enable a systematic approach to generate traceable and unique resource identifiers.

**Table 2:** Current key elements impeding proper tracking of bioresources use in scientific literature

***Difficulties related to identification and acknowledgement of bioresources***	Difficulties encountered with marker papers**
- multiplicity of sections where bioresources can be acknowledged (Material & Methods, Acknowledgements, References…)	- suitable to refer to one type of bioresource but not for any derived, or secondary bioresources
- bioresource acknowledgement or citation placed outside the title or abstract in the main paper (or in online supplementary materials) which can therefore only be detected via full-text mining and is not indexed in Pubmed or Web of Science	
- typing errors or approximation of the bioresource name/identification	
- multiplicity of names for a given bioresource	
- cascade use of resources (*e.g.* several CEPH* Family samples are part of the Hapmap which are themselves part of the 1000 Genome Project)	
- acknowledgement of persons instead of the bioresource itself	
-absence of acknowledgement for the bioresource used (negligence)	
- no standardized way to incentivise researchers to acknowledge properly the bioresource used	

^*^CEPH, Centre d’Etude du Polymorphisme Humain.

^**^[36].

With the purpose of addressing the various issues referred to above, bioresources need to be assigned actionable digital identifiers or IDs [18], and to fulfill the requirements of the scholarly record, the bioresource ID should be persistent, globally unique and citable. The BRIF ID sub-group focuses on exploring and assessing existing and emerging technical solutions suitable for bioresource identification, as well as addressing key related issues, such as what to identify (biobank projects, sample collections, databases, datasets, etc.) and which international and independent body (or bodies) should be responsible for assigning the bioresource ID.

The aim of BRIF is not to create a new identifier scheme specifically for bioresources, but rather to identify frameworks that are already established (or well on their way to becoming so), such as registries for clinical trial studies and other more general ID schemes [19], and to subsequently assess them and recommend their use as appropriate with respect to: i) resource providers (e.g., what type of IDs to use for biobank projects and databases); ii) end-users (e.g., editorial guidelines for authors on how to properly cite biobank projects and databases using unique resource IDs).

Findings of the sub-group are that the field is already moving in the expected direction in several areas; most notably, the DataCite initiative [20] has established a worldwide data registration agency that reuses and extends the Digital Object Identifier (DOI) scheme that is already widely used in the scholarly publishing domain [21,22]. Digital records describing bioresources (e.g., sample collections), or born-digital resources (datasets associated with a resource or generated by its use) can be assigned data DOIs [23]. This is an important step towards recognizing the creation and sharing of research data as a valuable scientific contribution [24]. DOIs could then serve as first-step identifiers to be used in the BRIF assessment. Other possibilities that are suitable for investigation include the World Health Organisation (WHO) [19], which is already involved in assigning clinical trials registration numbers, and the Organisation for Economic Co-operation and Development (OECD).

A closely-related issue is the current lack of global infrastructure for identifying and acknowledging researchers who contribute to bioresources [25]. The centralized contributor ID system now being built by the international Open Researcher and Contributor ID (ORCID) initiative [26] has been recently launched. It seems reasonable to assume that this emerging infrastructure will be at the heart of any attribution schemes for scientific contributions [27] and therefore likely to be very relevant for BRIF in the near future. Datacite and ORCID are now involved in a common European project, ODIN (the ORCID and DataCite Interoperability Network) that aims to design an ‘*awareness layer*’ for persistent author and object identifiers, notably by providing an Information Architect and Software Developer. It will explore how to connect the unique identifiers for persons and datasets across multiple services and infrastructures for scholarly communication.

#### Identifying and weighing parameters, measures and indicators

Establishing a valuable bioresource requires considerable time and effort, and in order to provide appropriate rewards and recognition, it is necessary to be able to measure their utility. There are various ways to do so such as using a range of indicators at various levels (biological samples, annotations and associated data, and search tools), including management indicators showing that the bioresource is efficiently run and well utilized; quality indicators such as the quantity of biospecimens available and the value of samples or datasets; and indicators of research productivity based on bioresource use and reuse. Since the purpose of the bioresources considered here is to enhance health research productivity, this last set of indicators may provide the most reliable assessment of appropriate biobank usage.

With these indicators in mind the BRIF parameters need to be both objective and easily verifiable, and the calculation of a BRIF needs to be as simple as possible. The main identified indicators have been grouped as follows (see Table 3): 1/age and size of the bioresource; 2/research productivity and sustainability (journals and papers, grants and patents, institutional funding); 3/sample/data value (follow up of data, diversity, rareness, quality control…); 4/bioresource management (workflow and efficiency); 5/networking and visibility of the bioresource. Downstream effects on healthcare and the economy will not be assessed. An obvious approach is to use a simple metric based on citation counts in conjunction with the traditional notion of journal-level impact. BRIF calculation will have to provide a measure of the extent to which bioresources contribute to research. Subsequent surveys are currently being done to identify and weight key parameters for the evaluation of bioresource impact in research, as well as to distinguish those that can be easily tracked and measured to construct the metrics of a meaningful BRIF.

**Table 3:** Range of indicators and parameters to take into consideration for BRIF

***First-line parameters***	***Second-line parameters***
**1)** Age of bioresource	
Size of bioresource	
**2) Indicators of research productivity:**	
- Quality of the journal (impact factor…)	- Grants obtained by the users of the bioresource or to support the bioresource
- Number of articles citing the bioresource itself or the staff	- Patents/licenses based on research supported by the bioresource
- Cumulated impact factor (or h index) of publications that result from research supported by the bioresource	- Economic impact
- Number of patents that result from the use of the bioresource	
- Distribution of samples having multiple involvement in independent projects…	
**3) Indicators of high value**	
- Rare disease samples or data / samples with rare characteristics	- Official recognition from Regional/National Health Bodies
- Extent and richness of the datasets collected	
- Existence of a quality control policy for samples and data	
- Compliance with data reporting nomenclatures and sharing standards	
- Participation in external assessment programmes such as certification or accreditation (ISO certification for example)	
- Availability of morphological controls of frozen specimens used for “omics” programme (biobanks)	
**4) Indicators of management**	
- Number of projects supported per year	- Number of samples received and distributed per year
- Number of biospecimens entering in the biobank / number of biospecimens used for distribution to research projects by year	- Number of material/data transfer agreements
- Number of requests filled per year (to be balanced with the type of resource)	- Number of contracts or agreements
- Number of web page accesses per year for data resources	- Average time from collection to actual use of the sample (sustainable maintenance)
	**Other factors:**
- Number of material (data) transfer agreements and contracts signed per year	- Return of research policy
- Turnaround time for requests	- Impact of data cost on inclination to correctly cite the source of data
- Time to include new data	- Past achievements of the bioresource…
- Consent forms	
- Data protection measures	
**5) Indicators of visibility**	
- Networks	
- Catalogues	
- General policies of transparency, dissemination, access rules…	

#### Analyzing the role of journal guidelines and policies for resource citing and referencing

A key element for assessing the use and the research impact of bioresources is via their systematic citation in journal articles. However, today there are no standards and guidelines for the citation of such resources. Even when authors make an honest attempt to properly acknowledge a bioresource, the results can be patchy and inconsistent. For example, the presence of the web address, or uniform resource locator (URL) of a database in biomedical papers provides some evidence of reuse; however, if the URL is not present in the abstract, then the reference to the database will not be discoverable via *PubMed* or other bibliographic databases which only index abstracts. Hence, journal publishers need to establish a clear policy concerning the citation and referencing of the contributing bioresource otherwise, measuring the impact of bioresources will remain an imprecise process.

Recognition by journal editors of the need to properly acknowledge the bioresources utilized, using proper terminology and/or identifiers and agreeing on standards of citation (format/marker paper, location(s), institutions, people, etc.) has been extensively discussed. The BRIF sub-group has initiated a dialogue with the Committee on Publication Ethics (COPE) and the International Committee of Medical Journal Editors (ICMJE) [28] in order to inform journal editors about the BRIF issues and to promote the modification of editorial guidelines accordingly. As “Uniform Requirements for Manuscripts Submitted to Biomedical Journals” [28] already exist, a first proposal has been sent to the ICMJE to be considered in a future amendment of these requirements. This proposal highlights some additional requirements (Table 4) that may be needed to address the editorial problems concerning bioresources. Furthermore, additional actions have been designed to sensitize other committees and institutions concerned with editorial and ethical issues. Notably, the European Association of Science Editors (EASE) is presently considering how to include citation of bioresources in their guidelines.

**Table 4:** **Main suggestions for the *Uniform Requirements for Manuscripts Submitted to Biomedical Journals: *Writing and Editing for Biomedical Publication** ( http://www.icmje.org)

***Guideline text section***	***Proposition***
*in* II. ETHICAL CONSIDERATIONS IN THE CONDUCT AND REPORTING OF RESEARCH	‘Biobankers should always be acknowledged for their contribution in providing "bioresources" useful for the conduct of the study. The name of the biobank (and identifier, if available) should also be reported here in full.’
II.A Authorship and contributorship	
II.A.2 Contributors listed in acknowledgements:	
*in* IV. MANUSCRIPT PREPARATION AND SUBMISSION	‘8. List of bioresources and/or biobanks used as sources of samples and/or data (and their identifier, if available). Bioresources include both biological samples with associated data (medical/epidemiological, social) and biomolecular research tools. The biosamples and biomolecular resources include any "physical" specimen derived from biological organisms, as well as antibody, affinity binder collections, clone collections, siRNA and microarrays libraries. Research tools include any data directly or undirectly derived from biosamples such as databases, locus specific-databases, registries of disease patients and any specific tool for molecular characterization of biobanked samples.’
IV.A Preparing a manuscript for submission to a biomedical journal	
IV.A.2 Title page	
	‘9. Infrastructures. National, European and/or international infrastructure that has evaluated the project.’

#### Assessing policies for resource access and sharing

Attempting to measure the impact of a bioresource is based upon the assumption that the research resource is actually being utilized. Use of a bioresource is contingent upon many factors, but the access and sharing policies certainly play a major role in facilitating or hindering use. Various components, such as the level of constraints imposed on users or the level of user-friendliness of the procedures to gain access, are pivotal to creating an environment that will stimulate or discourage using a given bioresource.

Appropriate indices to consider in implementing a strategy to measure and compare the impact of bioresources [29] include, sharing policies, access and publication policies and the agreements that support the ‘transaction’ of sharing material or data, as well as community standards, such as those indexed by BioSharing [30]. Through such guidelines or contracts, a bioresource can impose requirements on users that would enable the measure of its impact. Two dimensions are likely to contribute to the measure of the bioresource impact: dissemination and ‘control’ measures. Publications, academic presentations and other less traditional means of disseminating research results are critical. Bioresources must therefore ensure that users will recognize the resources that were used in whatever means the researchers use to communicate their results to the scientific community or to the public. This recognition must occur in such a way that will allow a systematic search to track use as described above.

Bioresources may also require users to report on their use back to the bioresource (e.g., sending their publication or a summary report). However, a balance must be struck between imposing a series of requirements on users and on bioresource managers and still maintaining conditions that foster resource use.

The level of control that the bioresource can exercise over the various secondary uses of its content is another factor that can enable the measurement of its scientific impact. In order for a bioresource to track the use of its content it must ensure that users comply with its dissemination requirements. This is particularly challenging for research databases where the data can be copied and circulated easily and *ad infinitum*. In a context where international collaboration is increasing and pooling of research resources is necessary to conduct research, for example on complex diseases and health, it is difficult for the bioresource to track all uses. The identity of the source of a material may be lost in the chain of multiple exchanges and amalgamation with others unless the link to a “mother resource” is traceable. A bioresource can thus require that users do not share the material/data with third parties. Under such circumstances, it is expected that users will have to deal directly with the initial bioresource provider to gain access, and will thus have the same requirements imposed upon them to recognize the original resources. Once again, a balance must be struck between imposing constraints on users and making use of the bioresource appealing. However, if the correct balance is reached, specific issues persist relating to databases where no physical entity is provided. To some extent, commercial data providers can impose constraints on the onward distribution of the data. A breach of corresponding terms and conditions might then allow the data provider to restrict future access. As to those databases that provide free access, large organizations can organize and support a number of control actions [29]. Small data providers, for example, curators of Locus Specific Databases for a small number of genes, have fewer opportunities to exert access control and simply rely on database copyright protection [31]. Given the delicate balance required between stimulating usage and supporting the capacity to measure the impact, the BRIF sub-group proposes to develop an appropriate set of standard tools that could eventually be integrated in the overall access and sharing policies of bioresources.

## Conclusions

### Perspectives: Current endeavors for practical development and implementation of BRIF

#### Pilot actions

To allow bioresource recognition to become rapidly entrenched in everyday research practices, it is essential to test the feasibility of the various aspects of the BRIF through several small-sized pilot studies each focusing on specific issues, such as the citation modalities, especially exploring the feasibility of a specific field for bioresources in electronic submission systems, the identifier entity, the authors compliance. This is being initiated with the help of volunteer consortiums (i.e., eagle-i [32], BioSHaRE [33], P3G [34], BiOBANQUES [35]), and being open to external proposals.

#### Outreach

The international outreach of the initiative is presently limited as an unbalanced geopolitical representation that has been mobilized so far in the BRIF working group. A dissemination and open access policy to the participation in this initiative is thus necessary and this paper aims to encourage this. Better geographical representation, contact with other networks and initiatives that could produce synergetic actions, and solicitation of international journal editors committees and institutional scientific evaluation boards involved in producing incentives and guidance towards researchers and authors may each contribute to better tailor the BRIF tool as required.

#### Metrics

Once a solid framework for bioresource research impact has been secured, the next step will be the actual production of a set of metrics and software to mine articles and bioresource information metadata in order to test which ones are best performing. More sophisticated factors would consider some measurements of bioresource quality and value, including origin of samples and their rareness that could also be further devised and integrated into the indexing system.

To address the need to incentivize the development, maintenance and sharing of bioresources, a set of principles, tools and guidelines is required. We conceptualized and formalized a framework for bioresources management, use and referencing on which the medical and scientific community could rely for their research practice. It can draw on technologies already in use for tracking and evaluation of impact in other science referencing areas. This article provides the foundations for the creation of the BRIF as an adequate instrument. It hopes to trigger discussion among relevant stakeholders and incite the scientific community to embark in this endeavor.

## Abbreviations

BRIF: Bioresource research impact factorCEPH familyCentre d’etude du polymorphisme humain family; DOI: Digital object identifier; ID: Digital identifier; ICMJE: International committee of medical journal editors; OECD: Organisation for economic co-operation and development; ODIN: ORCID and datacite interoperability network; ORCID: Open researcher and contributor ID; RE: Requirement engineering; URL: Uniform resource locator; WHO: World health organisation

## Competing interests

One of the reviewers is part of BioSharing [30], and has started to engage in the BRIF working group since the review. Please see the manuscript pre-publication history for further details.

## Authors’ contributions

JC and PH have been involved in contributing specifically to the introductory section of the article, GT and RD wrote the *‘Choosing adequate digital identifier schemes’* paragraph and other parts of the text, RH wrote the paragraph entitled ‘*Identifying and weighing parameters, measures and indicators’*, EB wrote the ‘*Analyzing the role of Journal guidelines and policies…*’, MD wrote the ‘Assessing *policies for resource access and sharing*…‘section. FK contributed to the definitions, the clarification of uses and provided examples. PAG, MAMF and MP provided examples in various contexts and contributed to the manuscript. MF contributed to the content of the tables and to other parts of the text. LM and ACT took the initiative of the paper, directed the BRIF initiative, wrote the parts related to the BRIF concept and objectives and the ‘Conclusion’ and ‘Perspectives’ paragraphs and proposed the content of the tables. JH contributed to the ‘*Assessing policies for resource access and sharing*’ section and shared with LM and ACT the charge of ensuring the global coherence of the manuscript; all authors reviewed and approved the whole text, tables and references.

## Authors’ information

ACT leads a multidisciplinary team on “Genomics, biotherapy and public health”, involving human and social sciences as well as health sciences, in the context of research in epidemiology and public health at Inserm (National Institute for Health and Medical Research) in Toulouse, France. She also leads a societal platform “Genetics and Society” at the Toulouse-Midi-Pyrénées Genopole. She is involved in several EU projects in genomic sciences, public health genomics and biobanks, much in relation to data sharing and open access management, where she is responsible of ethical, legal and social aspects (ELSA). She sits in several scientific advisory boards of international projects and is member of the scientific council of Inserm and of the board of the French and European Societies of Human Genetics. She is member of the Public and Professional Policy Committee of the European Society, and is involved in several ethics committees. Former member of the CCNE (French national advisory bioethics committee) and of the European Group on ethics of science and new technologies, she chairs the Life sciences operational ethics committee in CNRS (National Center for Scientific Research) and is a member of the deontology advisory committee of the National cancer institute. Besides her work in immunogenetics she has recently worked on societal aspects of biobanks focussing on data sharing and open access issues, biotherapies, human genetic and genomics testing and high throughput technologies. In the philosophy of the Data Sharing and Open Access endeavours, she created and leads the Bioresource Research Impact Factor (BRIF) initiative. This project started with the creation of an international BRIF working group which comprises of 134 members from 22 countries (http://www.gen2phen.org/groups/brif-bio-resource-impact-factor). LM is the BRIF project manager.
